# A digital PCR system based on the thermal cycled chip with multi helix winding capillary

**DOI:** 10.1038/s41598-020-74711-8

**Published:** 2020-10-20

**Authors:** Bin Li, Yuanming Li, Yangyang Jiang, Andreas Manz, Wenming Wu

**Affiliations:** 1 State Key Laboratory of Applied Optics, Changchun Institute of Optics, Fine Mechanics and Physics, Chinese Academy of Sciences, Changchun, 130033 China; 2 University of Chinese Academy of Sciences, Beijing, 100049 China; 3 Systems Engineering Department, Saarland University, 66123 Saarbrücken, Germany; 4 Bio Sensor & Materials Group, KIST Europe, 66123 Saarbrücken, Germany

**Keywords:** Biological techniques, Molecular biology

## Abstract

This paper presents a digital PCR system based on a novel thermal cycled chip, which wraps microchannels on a trapezoidal structure made of polydimethylsiloxane (PDMS) in a multi-helix manner for the first time. It is found that compared to the single helix chip commonly used in previous reports, this kind of novel multi-helix chip can make the surface temperature in the renaturation zone more uniform, and even in the case of rapid fluid flow, it can improve the efficiency of the polymerase chain reaction. What’s more, the winding method of multi helix (such as double helix, six helix and eight helix) can obtain better temperature uniformity than the winding of odd helix (such as single helix and three helix). As a proof of concept, the temperature-optimized double-helical chip structure is applied to continuous-flow digital PCR and there is no need to add any surfactant to both the oil phase and reagent. In addition, we successfully analyzed the fluorescence signal of continuous-flow digital PCR by using CMOS camera. Finally, this method is applied for the absolute quantification of the clinical serum sample infected by HBV. The accuracy of the test results has been confirmed by commercial instruments.

## Introduction

Polymerase chain reaction (PCR) is a nucleic acid molecular cloning technique. It can amplify nucleic acid molecules from one to several million in a very short period of time (usually in tens of minutes), so it is regarded as the most important molecular biology technology today. It plays an important role in the fields including next-generation high-throughput sequencing and clinical nucleic acid marker detection^1–14^.

In 1998, Manz first proposed a new method to realize PCR through snake-shaped reciprocating pipeline in the world. Compared with the traditional temperature control technology based on the Peltier effect, this "chip laboratory" based expansion technology can make the temperature rise and fall faster. In recent years, this technology has been widely used in biology and medicine.

In the past decades, continuous-flow PCR technology^15–18^ has been successfully applied to all the fields of traditional PCR, real-time quantitative PCR and digital PCR^16,19–24^. Especially in recent years, the introduction of continuous-flow PCR based on absolute quantification of nucleic acid by digital droplets^25–30^ has greatly expanded the application prospects of this technology, and has broken the limitation of traditional methods to achieve digital PCR by relying on droplet array^31–34^ and temperature control mode of Peltier effect^35–39^.

The reason why the temperature rises and fall of the continuous-flow PCR technology is faster than the traditional Peltier effect is that the chip structure transforms the thermal cycle in time into that in space.

It is well known that thermal cycle is the core of PCR and one of the decisive factors affecting the efficiency of PCR. Different from the temperature control mechanism using the Peltier effect, chip design and geometric structure optimization are the key to improve the efficiency of continuous flow PCR. However, for the previous papers, we find that the design ideas of continuous-flow PCR chips are all single-helix or single-cycle reciprocating microchannel design schemes, the topology of which is relatively simple. This leads us to speculate whether the use of Micro-Pipes with relatively complex topologies (such as multi-helix structures) in continuous-flow PCR can make the control of thermal cycle better, although such complex structures have rarely been reported in previous studies of PCR applications.

The digital PCR is currently the only technology that can achieve absolute quantification of accounting, and it is also considered to be the most sensitive nucleic acid detection method. Currently, many methods have been developed for performing continuous-flow PCR reactions. The development of droplet-based microfluidics has completely changed the dispersion process of PCR samples. It can reduce the droplet volume to attoliters, which greatly reduces the possibility of contamination, increases the flux of digital PCR and reduces the possibility of multi template nucleic acid. In 2015, PDMS microfluidic chip was used for droplet digital PCR to generate highly monodisperse droplets using pressure difference. In 2017, N. Reginald Beer et al. designed and developed a real-time droplet PCR device based on T-channel chip. The PCR reaction was carried out in a water-in-oil droplet with an average volume of 13 picoliter. The syringe pump is used to deliver liquid to the device^40^. These methods include the engraving or copying of microchannels onto a plate and being wound on multiple heaters^41–43^, and these methods have proven useful for the detection of DNA amplification results.

However, due to the lack of ability to track droplets during the reaction, the above method can perform quantitative and real-time characterization of digital PCR using a two-phase fluid emulsion in a continuous-flow device, but fluorescence detection can only be performed at the end of the microchannel. In addition, traditional continuous-flow digital PCR often require multiple heater with constant temperature to achieve extended denaturation, renaturation and elongation temperatures.

The above discussion shows that the control of thermal cycle is the core of digital PCR. However, the traditional continuous-flow digital PCR has a series of drawbacks such as complex temperature control and failure to realize real-time detection and analysis. In order to solve these problems, our system uses micro-pipes to wrap a special structure to achieve PCR amplification using a single heater. This work provides a new type of dPCR device that expands the detection range of droplet fluorescence, reduces the total power consumption of the thermal cycler, and reduces the size of the device itself. What's more, in our system, the combination of an LED excitation source and a Complementary Metal Oxide Semiconductor (CMOS) camera detects the real-time fluorescence of each droplet from start to finish. The micro-pipes use a special multi-helix winding method, which can directly compare the fluorescence brightness of different cycle numbers while observing the fluorescence of the droplets.

## Methods

### Microfluidics chip

**Figure 1:**
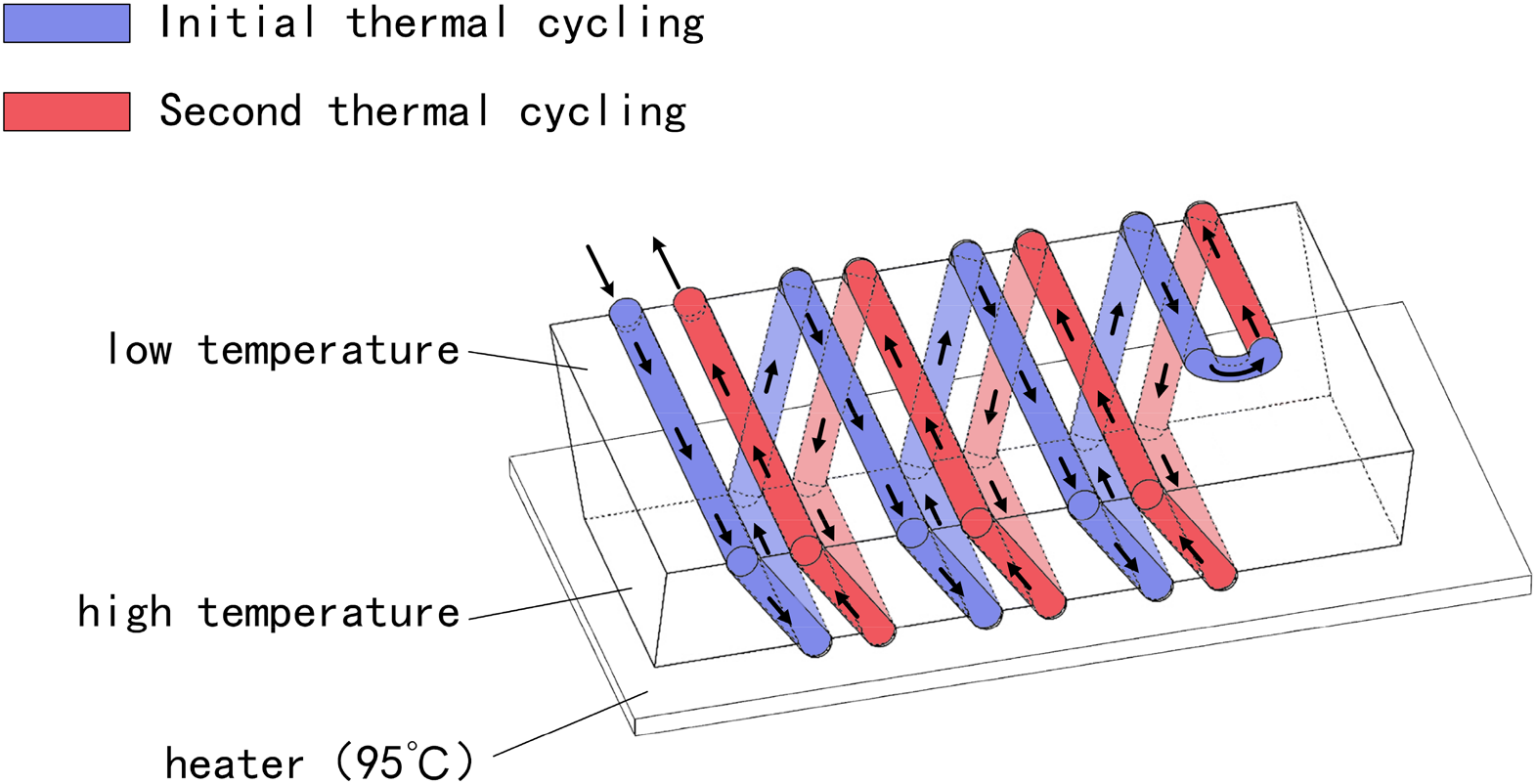
3D microfluidic chip in the form of double helix.

The Teflon micro-tube was wound on a trapezoidal structure made of polydimethylsiloxane (PDMS) as a chip for continuous-flow polymerase chain reaction. If the chip is placed on a constant temperature heat source, a temperature gradient can be formed on the upper and lower surfaces of the chip by natural convection of the air, thereby realizing a process in which the reaction chemical agent undergoes repeated temperature rise and fall while continuously flowing. The geometric configuration mode adopts a helix composite chip configuration in the form of double helix (Fig. 1), three helix, four helix, five helix, and six helix. Here, the single helix structure: Teflon tube (ID: 0.15 mm, OD: 0.3 mm) are wound from one end of the structure to the other, forming a path for fluid to pass through. Double helix structure: Teflon microtubules are bent after winding from one end of the structure to the other end, and continue winding around the structure until they return to the initial end of the structure. The composite chip formed by bending microtubules once is called double helix chip, and the composite chip obtained by bending microtubules twice is called triple helix chip. Such a configuration compensates for the inconspicuous separation of droplets in a single helix winding manner and the interference with heater temperature uniformity. Using a single helix winding method, the micro-pipes of the first ring and the micro-pipes of the last circle are respectively located at the two ends of the chip, and are separated by a distance in the spatial distance, so as to observe between the first-circle micro-pipe and the last-turn micro-pipe. It is difficult to accurately recognize the fluorescence brightness when the fluorescence changes. In extreme cases (a. no nucleic acid molecules in the reaction chemical reagent; b. a large number of accounting molecules in the reaction chemical reagent), the number of nucleic acid molecules in each digital droplet is zero or more than one, all The droplets produce the same fluorescent signal on the last lap of the reaction, and the reason is that the extremes cannot be judged, which can bias the detection of the digital polymerase chain reaction. In addition, for a single-helical wound polymerase chain reaction chip, the cyclic reaction of the chemical reaction reagent from the high temperature region of the lower surface to the low temperature region of the upper surface flows in a single direction clockwise or counterclockwise, which causes the upper surface The entire plane of the low temperature zone produces a temperature gradient of more than 10 °C in this direction, so that the annealing temperature and the extension temperature are not constant, which greatly reduces the reaction efficiency.

The configuration of multi-helix wound composite chip solves the above problems well. The winding mode of multi-helix will cause a gap in the number of cycles between adjacent microchannels, and the more the number of helixes, the less the difference in the number of cycles between adjacent microchannels. Therefore, in fluorescence analysis, in the same position, fluorescence can be analyzed in multiple pipelines with large difference in the number of cycles. While reducing the fluorescence error, we can better analyze the fluorescence change trend from the first to the last circle, which makes it easier to analyze the extreme cases of chemical reaction reagents which contain no nucleic acid molecules or a large number of nucleic acid molecules. The geometry of the multi-helix chip mentioned above is also helpful to solve the problem of temperature variation in the low temperature region. The reason why multi-helix winding can improve the temperature stability in high and low temperature zone is that it avoids the disadvantage of chemical reagents flowing in one direction between micro-fluidic pipes. Chemical reagents flow in two directions between adjacent pipes. The flow direction between adjacent pipes meets both the flow direction from high temperature zone to low temperature zone and from low temperature zone to high temperature zone. The two characteristics of flow make use of heat conduction between adjacent pipes to achieve temperature balance, which greatly improves the temperature stability of the whole region. Therefore, these methods have significant advantages for rapid polymerase chain reaction. The temperature of the upper surface of the multi-helix wound chip can be kept in balance even when the fluid flows rapidly, and no surfactant is needed during the whole experiment. Under the same circumstance, the temperature difference of the upper surface will become more obvious with the increase of the flow rate, and even lead to the failure of the reaction.

### Droplet generation

**Figure 2:**
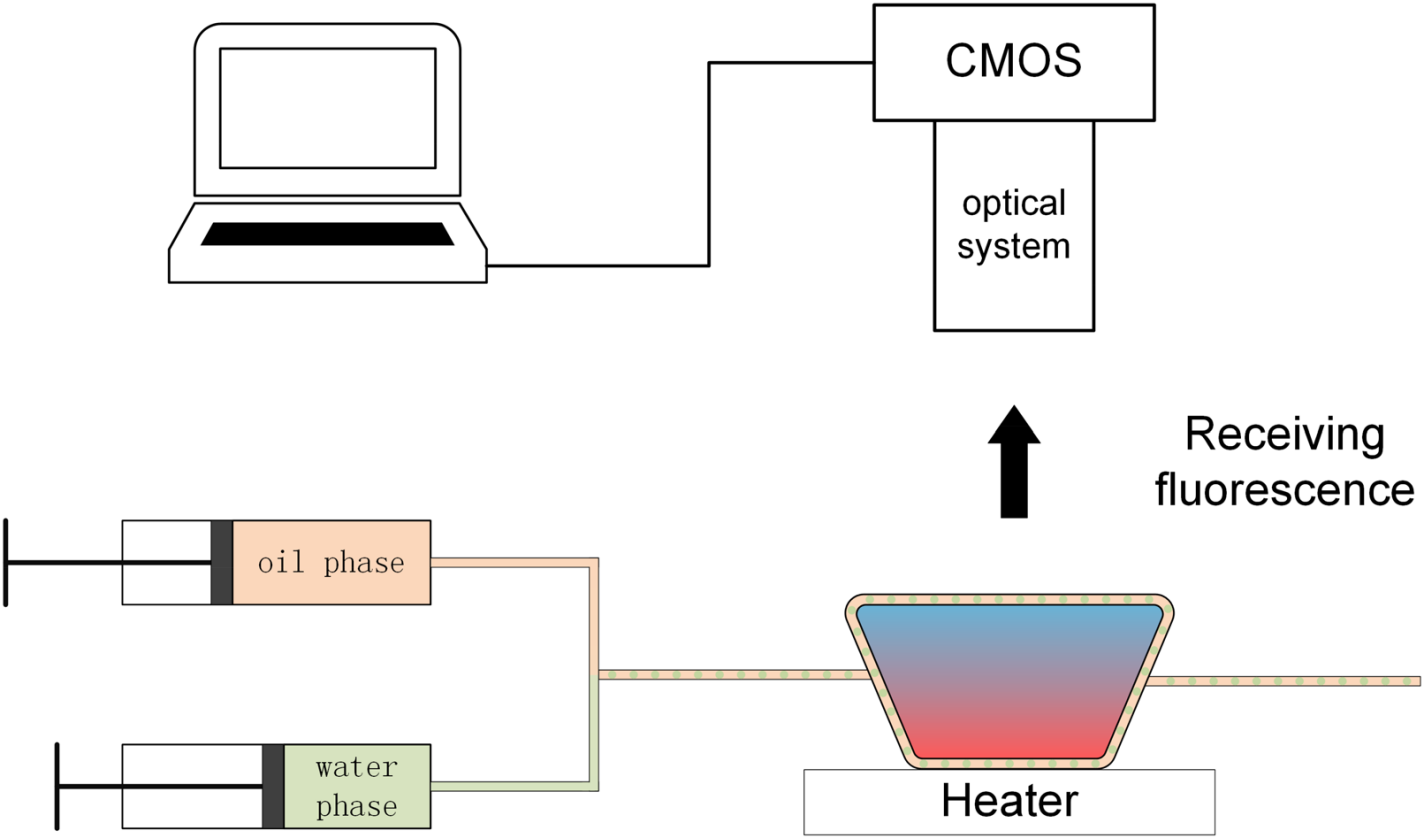
Schematic of droplet generator.

As shown in Fig. 2, the droplet generating device is composed of two 34 g needles connected by a locally stretched Teflon capillary, and the other end of the needle is connected with the water and oil phases of the syringe. The syringe is powered by the syringe pump, so that the water droplets are trapped in the immiscible oil phase and introduced into the Teflon capillary. By controlling the propulsive speed of the water phase syringe pump, the monodisperse droplets with specific size can be produced, and the moving speed of the droplets can be controlled by controlling the oil phase syringe pump. The carrier fluid used in all experiments is fluoride.

### Thermocycling system

The thermocycling system consists of two parts: a heater providing suitable temperature for amplification and a trapezoidal 3D microfluidic chip. PDMS was used to fabricate quadrilateral structure with isosceles trapezoidal cross section. Teflon (4 times stretched) microtubules were wound around the structure in the form of multi-helix to form a trapezoidal 3D microfluidic chip. The heater contacts the topline of the trapezoidal chip, and the contact part is the high temperature zone. A silicon wafer is placed between the heater and the topline of the trapezoidal chip to make the heating uniform. The topline of trapezoidal chip is high temperature denaturation zone, and the baseline of trapezoidal contacts are low temperature annealing and elongation zone. The capillary is wound around the structure and placed on a heater to provide the temperature required for denaturation, annealing and elongation of the PCR process. For the experiments discussed here, we choose 95 °C (+ 0.5 °C) as denaturation temperature and 58.6 °C (+ 0.5 °C) as annealing and elongation temperature. The time of each cycle is determined by the diameter of the microchannel and the velocity of the oil phase carrying the droplets.

### Reagents

Hepatitis B virus nucleic acid detection kit (fluorescent probe method) used in the experiment was provided by NEPG Liaoning Bio-pharma Co., Ltd. The reaction reagent was prepared according to the instruction manual. The total amount of reagents prepared was 50 μl, including 18 μl HBV PCR reaction mixture, 2 μl PCR enzyme mixture and 30 μl HBV DNA template. The DNA template was obtained by magnetic bead extraction from standard HBV samples. 8 μl reagent was injected into the syringe of water phase in each experiment. The thermal cycle adopts two-step method, and the temperature condition is set as follows: 95 °C for 10 s and 60 °C for 30 s.

### Image acquisition and processing

The fluorescence detection system uses 3 W high-power LED as the fluorescence excitation light source, and 480 nm filter is installed in front of the excitation light source to ensure that it can excite fluorescence, but it will not generate excessive noise. The fluorescence receiving device uses CMOS camera with 20 million pixels. The front end of CMOS camera is equipped with optical lens and 520 nm filter to receive the fluorescence signal and display the real-time image on the PC connected with it. Because the optical lens can adjust the focal length according to the need, this structure can monitor the fluorescence of each cycle of the amplification reaction in real time. The fluorescent droplet counting software installed on PC will calculate and count the brightness of each droplet by playing video on the software interface, and then draw the brightness map of the droplet through the derived Excel table to get the linear relationship.

## Results and discussion

**Figure 3:**
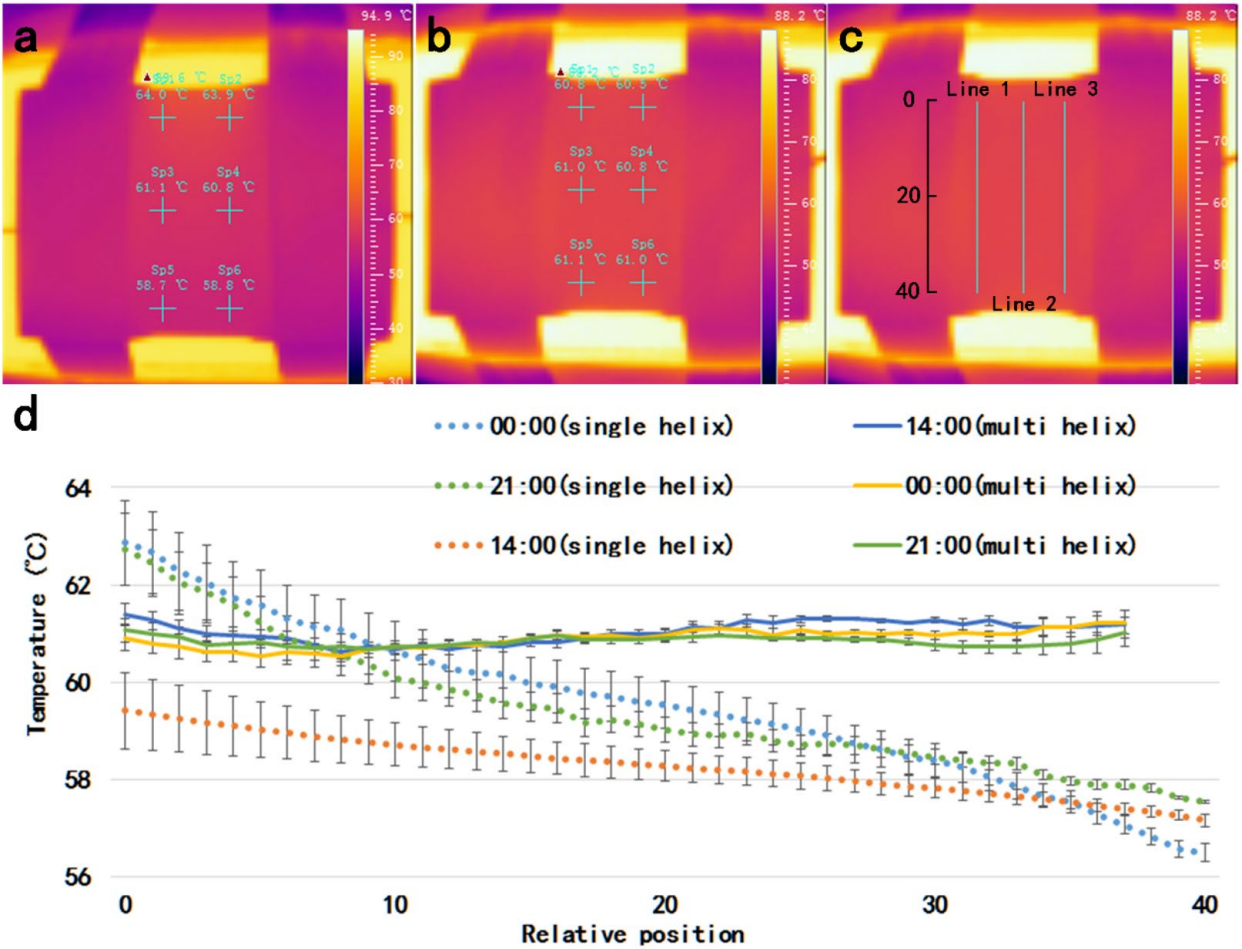
(**a**) Temperature uniformity of single helical winding mode; (**b**) temperature uniformity of double helical winding mode; (**c**) line segment selection diagram for line temperature measurement; (**d**) line temperature distribution curve with standard error at different time.

Microfluidic chips fabricated with the same structure are heated on the heating table at the same flow rate. High-resolution infrared thermal imager (Fotric 220, ZXF Laboratory, TX) is used to collect temperature information in the low temperature region, and the thermodynamic diagram obtained is shown in Fig. 3. In both experiments, 6 observation points were selected for the cryogenic zone of chip, and the average temperature of these observation points was about 61 °C by adjusting the temperature of heating station. It is obvious that the temperature of the single helix winding mode is not uniform on the plane of the thermal imager. In the six observation points, the transverse two points are 1 °C different, while the longitudinal three points are 3 °C different, and the maximum temperature is 6 °C °C different from the minimum temperature (Fig. 3a). In contrast, the temperature of the chip with double helix winding mode is relatively uniform on the plane of the thermal imager. In the six observation points, the difference between the two transverse points is 0.1 °C–0.3 °C, the difference between the three longitudinal points is 0.1 °C–0.3 °C, and the difference between the highest temperature and the lowest temperature is 0.5 °C (Fig. 3b). As shown in Fig. 3c, three lines are set on the thermal image to test the linear uniformity of chip surface temperature, and a ruler is set vertically to indicate the relative position. From the linear temperature uniformity of different heating time, the linear temperature of single helix chips is very uneven. The temperature difference is about 7 °C, although the temperature becomes uniform gradually with the heating time increasing, the temperature difference is still maintained at about 2 °C. The temperature of the chip in the double helix mode is relatively uniform on the whole, and the temperature difference is maintained within 1 °C no matter how long it is heated. The winding mode is set as a unique variable by controlling variable method, which proves that the double helix structure has better ability to maintain surface temperature uniformity than the single helix structure, and the temperature of the chip reaction zone made by the double helix structure is more uniform.

**Figure 4:**
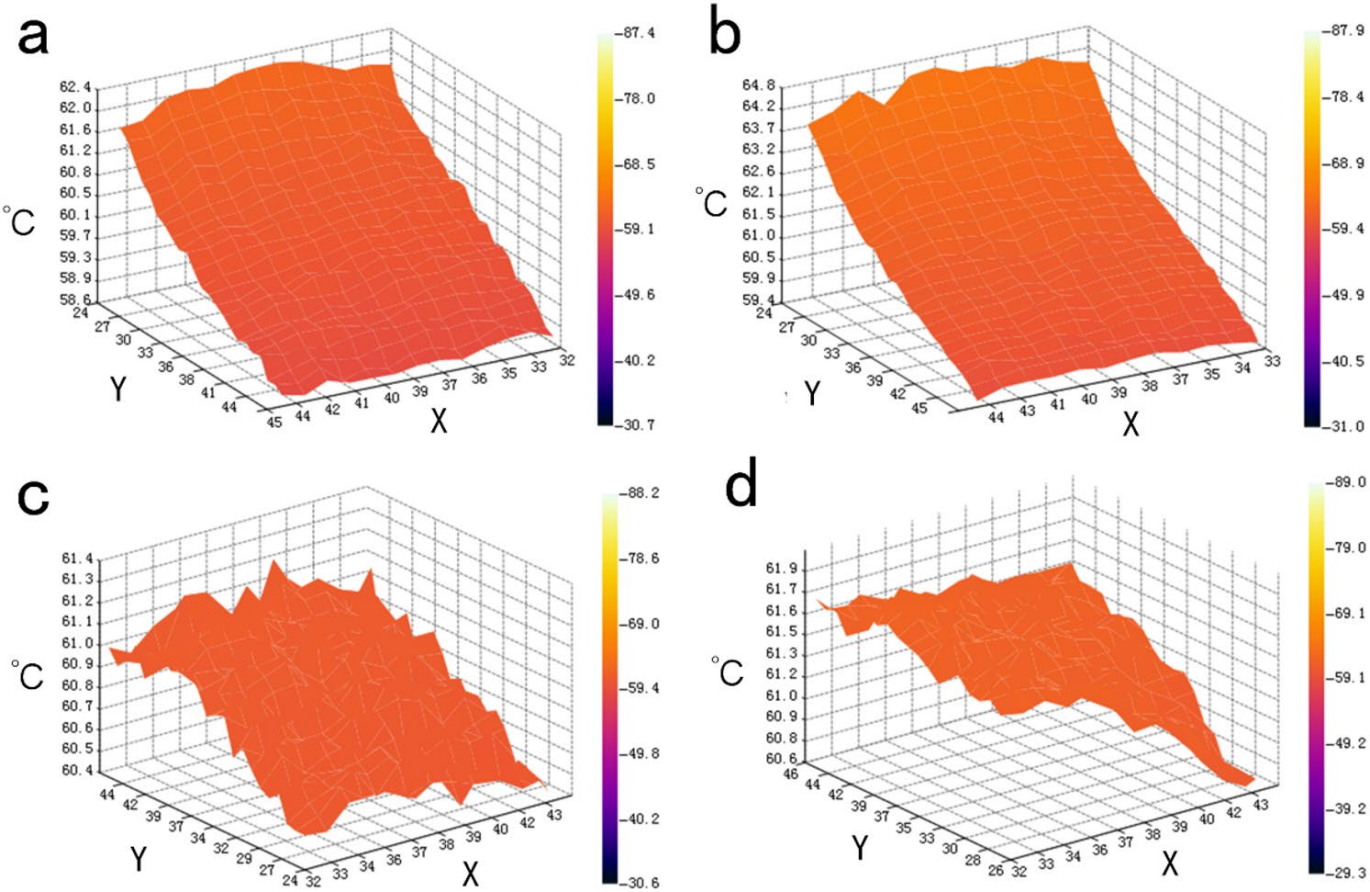
Temperature distribution in low temperature zone of chip. (**a**) When the velocity of flow is 2 ml/h, the temperature distribution in single helix mode; (**b**) When the velocity of flow is 3 ml/h, the temperature distribution in single helix mode; (**c**) When the velocity of flow is 2 ml/h, the temperature distribution in double helix mode; (**d**) When the velocity of flow is 3 ml/h, the temperature distribution in double helix mode.

In order to observe the temperature uniformity in the low temperature region more intuitively, we extend the temperature detection range to the whole plane of the low temperature region. The chips with the rates of 2 ml/h and 3 ml/h of the reagents were compared. (Fig. 4) We find that the temperature of the single helix winding mode decreases rapidly along the direction of reagent flow in the microchannel, and the difference of temperature is about 4 °C when the rate is 2 ml/h (Fig. 4a), and 5 °C when the rate is 3 ml/h (Fig. 4b). The temperature of the double helix winding mode will also decrease along one direction, but the difference of temperature is relatively small, regardless of the rate, it is 1 °C. (Fig. 4c,d), compared with single-helix winding mode, the temperature of double-helix winding mode is more uniform, which can make the temperature of microchannels with different cycles close to the same, which is conducive to the success of the experiment.

**Figure 5:**
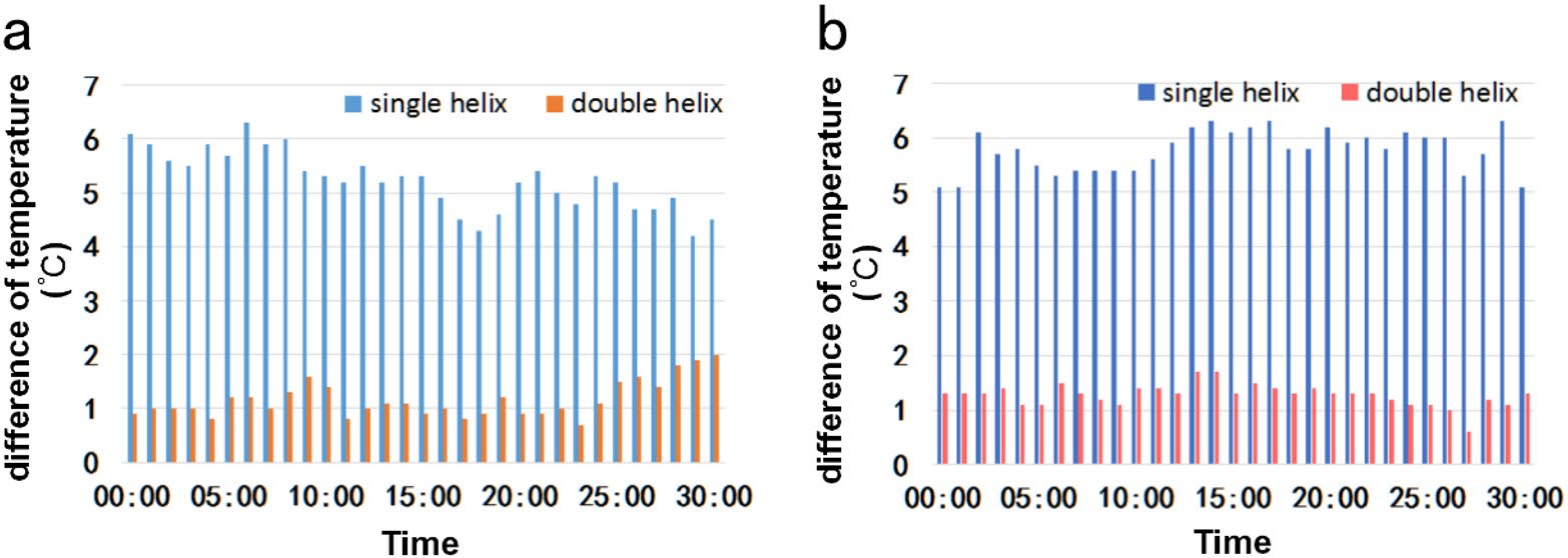
The change of difference between the lowest temperature and the highest temperature in the selected area with time goes by. (**a**) The change of difference of temperature when the velocity of flow is 2 ml/h; (**b**) The change of difference of temperature when the velocity of flow is 3 ml/h.

Temperature uniformity in the region is far from enough. Continuous-flow PCR also requires that the temperature is stable before the amplification was completed. As in the above experiment, the reagent in the micro-tube flow continuously for a period of time, and record the temperature of the observation area with infrared thermal imager. As shown in Fig. 5a, when the velocity of flow is 2 ml/h, the difference of temperature in single helix mode is maintained at 4 °C to 6 °C. And the difference of temperature in single helix mode increases obviously when the velocity of flow is 3 ml/h, which maintained at 5 °C to 7 °C (Fig. 5b). But in double helix mode, the difference of temperature maintained at 1 °C to 2 °C all the time regardless of winding mode. The results show that the double helix model can keep the temperature of chip stable for a long time.

**Figure 6:**
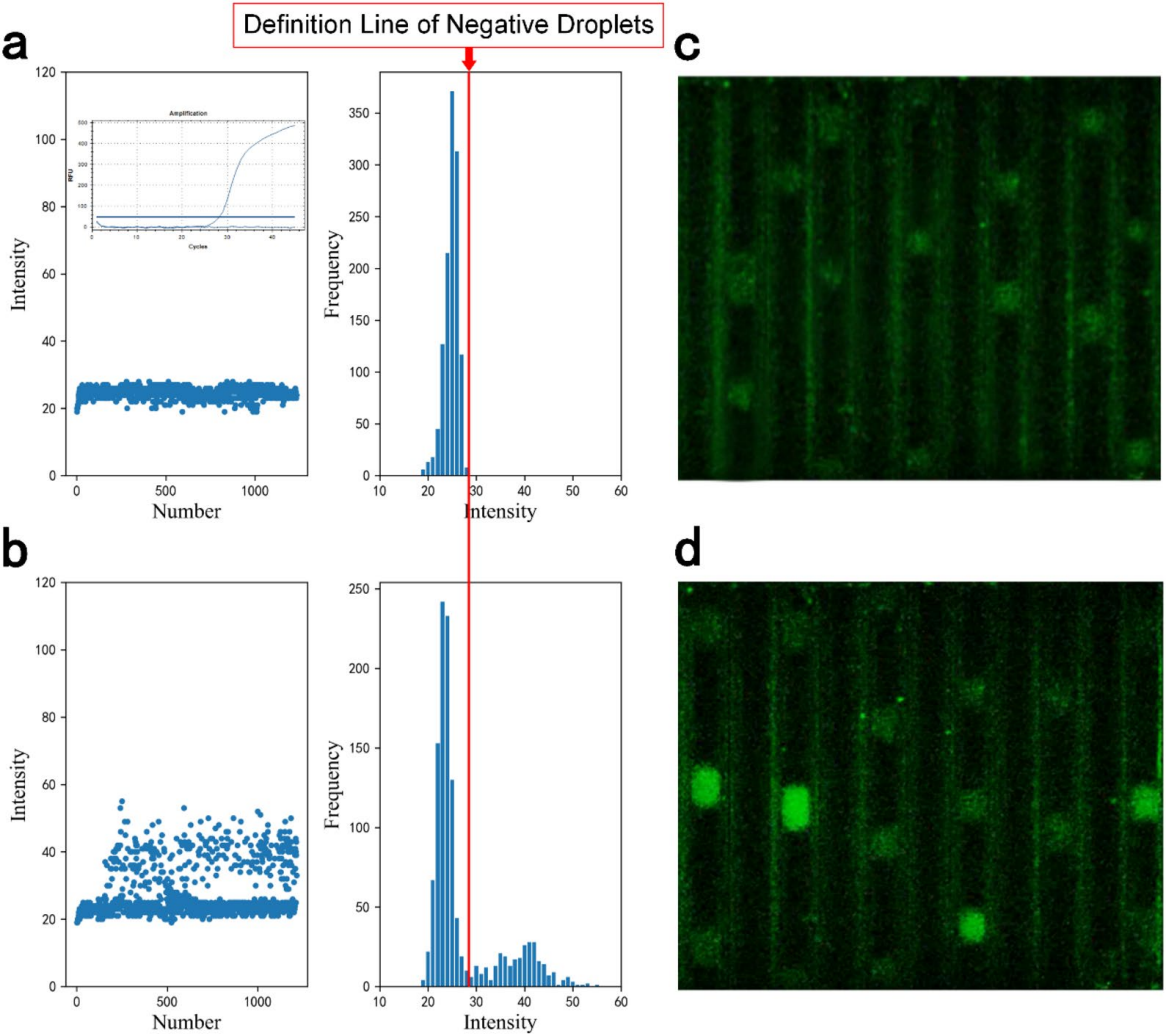
(**a**) Initial brightness of droplets; (**b**) Brightness of droplets after amplification; (**c**) Real-time fluorescence image of droplets of negative control group; (**d**) Real-time fluorescence image of droplets of experimental group.

As a proof of concept, this novel double-helix chip system can be used in continuous-flow PCR to detect clinical serum samples. In order to be able to count the number of droplets more accurately, we designed an automatic image recognition and processing analysis software for counting the number of droplets in a video shot in CMOS camera. The video is imported into this software for contrast and brightness modification, making it easier to distinguish between low-brightness droplets and high-brightness droplets. In order to determine the brightness threshold of digital PCR negative droplets, we first analyzed the fluorescence of the negative control group. The brightness of the droplets is counted as low brightness and the data is represented in Fig. 6a as a criterion for distinguishing between high brightness and low brightness. Just by selecting an area on the pipe whose reagent has not been reacted, and the software automatically records the relative brightness value of the corresponding pixel in the display, which can be used as the background brightness. In order to make the experiment more comparative, then the fluorescence data collected from the experimental group were analyzed, and the brightness of the droplet is counted again and output as Excel data (Fig. 6b). Figure 6c is the real-time fluorescence image of negative control group. Figure 6d is a real-time fluorescence image of the sample (concentration: 10^4^ IU/ml) taken by CMOS camera. The results detected by commercialized qPCR are also shown in Fig. 6a displaying a same result with the dPCR. It is obvious that high-intensity droplets are distinguished from low-intensity droplets and are easily resolved.

## Conclusion

Experiments show that this new multi-helix geometric structure as firstly reported in this paper has higher temperature stability in the process of single heater driven thermal cycle. Based on the optimization of the thermal cycle process, we have realized the absolute quantification of clinical serum samples (HBV) by continuous-flow digital PCR based on a single heater for the first time in the world. The use of CMOS camera is also reported firstly in this paper for continuous-flow digital PCR system instead of the probing sensor in traditional work flow, which raises the fluorescence observation range to several times the end detection observation range, and can distinguish the extreme cases where all droplets contain detection fragments or do not contain detection fragments, so as to make the detection more accurate. In addition, As far as we know, this is also the first time in the world that continuous-flow digital PCR without surfactant was realized. No surfactant was used during the whole experiment, which reduced the operation steps and the probability of errors in reagent configuration.
